# Effects of Biogas Slurry Combined With Chemical Fertilizer on Soil Bacterial and Fungal Community Composition in a Paddy Field

**DOI:** 10.3389/fmicb.2021.655515

**Published:** 2021-08-30

**Authors:** Hanlin Zhang, Shuangxi Li, Xianqing Zheng, Juanqin Zhang, Naling Bai, Haiyun Zhang, Weiguang Lv

**Affiliations:** ^1^Eco-environmental Protection Research Institute, Shanghai Academy of Agricultural Sciences, Shanghai, China; ^2^Agricultural Environment and Farmland Conservation Experiment Station of Ministry Agriculture, Shanghai, China; ^3^Shanghai Key Laboratory of Horticultural Technology, Shanghai, China

**Keywords:** paddy soil, biogas slurry, chemical fertilizer, bacterial community, fungal community

## Abstract

The application of biogas slurry and chemical fertilizer in paddy fields can be a practical method to reduce the environmental risk and utilize the nutrients of biogas slurry. The responses of bacterial and fungal communities to the application of biogas slurry and chemical fertilizer are important reflections of the quality of the ecological environment. In this study, based on a 3-year field experiment with different ratios of biogas slurry and chemical fertilizer (applying the same pure nitrogen amount), the Illumina MiSeq platform was used to investigate the bacterial and fungal community diversity and composition in paddy soil. Our results revealed that compared with the observations under regular chemical fertilization, on the basis of stable paddy yield, the application of biogas slurry combined with chemical fertilizer significantly enhanced the soil nutrient availability and bacterial community diversity and reduced the fungal community diversity. Dissolved organic carbon (DOC), DOC/SOC (soil organic carbon), available nitrogen (AN) and available phosphorus (AP) were positively correlated with the bacterial community diversity, but no soil property was significantly associated with the fungal community. The bacterial community was primarily driven by the application of biogas slurry combined with chemical fertilizer (40.78%), while the fungal community was almost equally affected by the addition of pure biogas slurry, chemical fertilizer and biogas slurry combined with chemical fertilizer (25.65–28.72%). Biogas slurry combined with chemical fertilizer significantly enriched *Proteobacteria*, *Acidobacteria*, *Planctomycetes*, *Rokubacteria*, and *Ascomycota* and depleted *Chloroflexi*, *Bacteroidetes*, *Crenarchaeota*, *Basidiomycota*, and *Glomeromycota*. The observation of the alteration of some bacteria- and fungus-specific taxa provides insights for the proper application of biogas slurry combined with chemical fertilizer, which has the potential to promote crop growth and inhibit pathogens.

## Introduction

With the rapid development of modern agriculture, much agricultural waste has been produced. Biogas projects are considered to be an effective method for the anaerobic digestion of animal manure or crop straws and have been widely used (Abubaker et al., 2012; Wang et al., 2019). In China, 110,473 biogas projects (including 6,972 large-scale biogas projects) were performed in 2016, and 139,295 biogas projects (including 10,122 large-scale biogas projects) are expected to be implemented in 2020 (National Development and Reform Commission and Ministry of Agriculture and Rural Affairs of the People’s Republic of China (NDRCMARAPRC), 2017). Biogas projects effectively solve the problem of agricultural waste recycling, but a large amount of biogas slurry is produced as a byproduct. Biogas slurry entering the environment without proper treatment can cause serious water and air pollution (Nkoa, 2014; Insam et al., 2015).

In addition to its associated environmental risk, biogas slurry is rich in nutrients and trace elements and can be used as a kind of high-quality organic fertilizer that is generally applied directly or combined with other fertilizer (Möller and Müller, 2012; Baral et al., 2017). Previous studies have shown that appropriate use of biogas slurry can increase soil nutrients, enhance crop nutritional quality, reduce greenhouse gas emissions, decrease crop disease and have other benefits (Terhoeven-Urselmans et al., 2009; Louro et al., 2013; Wang L. et al., 2018; Xu M. et al., 2019). However, the excessive application of biogas slurry will also cause some negative effects, such as the pollution of surface water and groundwater due to excessive nutrient input, the risk of heavy metal and organic pollutant accumulation in the soil, and the reduction of crop yield (Bian et al., 2015, 2016; Zhang et al., 2017; Cheng et al., 2018). Therefore, it is important and necessary to explore appropriate methods for biogas slurry application in agricultural systems.

Bacterial and fungal community diversity is a widely used ecological indicator that can reflect the quality of the ecological environment (Bell et al., 2012; Gonthier et al., 2014). Some previous studies have shown that the use of biogas slurry can significantly affect the soil microbial community. Xu M. et al. (2019); Xu Z. et al. (2019) found that a moderate dose of pure biogas slurry application could positively affect the soil bacterial diversity in rice-rape rotation systems, with too much or too little application showing the opposite result. The results of Abubaker et al. (2012) showed that biogas residues increased the microbial activity in wheat soil. However, Wentzel et al. (2015) observed that the application of biogas slurry as fertilizer reduced soil microbial activity and that the ratio of fungal C to bacterial C decreased with increasing soil clay content. In an incubation experiment performed by Johansen et al. (2013), digested materials only slightly altered the soil microbial community composition. The effect of biogas slurry addition on the soil microbial community was not consistent and depended on various factors, such as the application method, usage dose, soil type, and crop type. Bacteria and fungi strongly interact and cooperate with one another in the soil nutrient cycle, and they play important roles in ecological function (Liu et al., 2016; Wang H. et al., 2018). Although much research has been performed, few reports have addressed both bacterial and fungal community alterations following biogas slurry application. It is helpful to optimize and enhance the application of biogas slurry by studying the effects of different measures on soil bacterial and fungal communities.

In order to realize the ecological treatment of a large amount of biogas slurry, using biogas slurry to irrigate paddy fields can meet the needs of rice growth in terms of water and a large number of nutrient elements. It can also facilitate the reduction in the treatment of and enhance the resource utilization of biogas slurry. Therefore, biogas slurry is widely used as an organic fertilizer in paddy fields in China. We hypothesized that there will be one ratio between biogas slurry and chemical fertilizer which exerts more suitable impact on the growth of bacteria and fungi than the others, thus changing soil nutrient cycling efficiency and crop production. In this study, based on a 3-year field experiment involving the application of biogas slurry combined with chemical fertilizer in a rice-fallow rotation system, the Illumina MiSeq approach was employed to investigate the associated bacterial and fungal community diversity and composition. The aims of this study were to (1) identify the shifts in bacterial and fungal community diversity and composition, (2) associate the functions of particular taxa with specific biogas slurry application approaches, and (3) under the premise of a stable or increasing yield of rice, provide scientific support for the efficient and environmental utilization of biogas slurry. The results of this study may be useful for efficient recycling of biogas slurry and ecological paddy production.

## Materials and Methods

### Site Description and Experimental Design

The experimental area of this study is located at the Shanghai farm, Dafeng city, Jiangsu Province (33°32′ N, 120°54′ E). The annual average temperature and precipitation in this area are 14 degrees and 1,042 mm, respectively. The annual sunshine duration is 2,239 h. The soil was derived from a reclaimed yellow beach, and its type is sandy loam. Soil properties before the experiment was conducted included a pH of 8.71, soil organic carbon (SOC) of 8.36 g/kg, total nitrogen (TN) of 1.02 g/kg, and total phosphorus (TP) of 0.89 g/kg.

A rice-fallow rotation system was adopted in the experimental area. The experiment began in the rice season of 2015 and ended after the rice harvest in 2017. Six treatments were performed in triplicate under a randomized block design: regular chemical fertilizer treatment (RT, with pure chemical fertilizer application), biogas slurry replacing 25% chemical fertilizer (BS25), biogas slurry replacing 50% chemical fertilizer (BS50), biogas slurry replacing 75% chemical fertilizer (BS75), biogas slurry replacing 100% chemical fertilizer (BS100) and no fertilizer control (CK). The fertilizer dose applied during the rice season was the same in every treatment and based on the local regime, which was pure nitrogen at 225 kg/ha, phosphorus at 16.4 kg/ha, and the application was repeated every year (Table 1). The area of one replicate was 833 m^2^, and the replicates were separated by a soil ridge with an impermeable membrane.

**TABLE 1:** Amounts of fertilizer and biogas slurry applied in the experimental area.

	**Biogas slurry t^.^ha^–1^**	**Urea kg^.^ha^–1^**	**Ca(H_2_PO_4_)_2_^.^ H_2_O kg^.^ha^–1^**	**Total N kg^.^ha^–1^**	**Total P_2_O_5_ kg^.^ha^–1^**
CK	0	0	0	0	0
RT	0	490	500	225	75
BS25	49	367.5	485	225	75
BS50	98	245	470	225	75
BS75	147	122.5	455	225	75
BS100	196	0	440	225	75

The biogas slurry originated from the pig manure anaerobic fermentation tank on the Shanghai farm and was stored and precipitated in the storage pool. The supernatant of the biogas slurry entered the paddy field through the cement irrigation ditch with irrigation water. The total water volume was also consistent for each treatment. The properties of the biogas slurry were determined and calculated for each application. The average values of the applied biogas slurry were pH: 7.96 ± 0.12, TN: 1501.4 ± 82.5 mg/L, NH_4_^+^-N: 1378.6 ± 71.1 mg/L, TP: 62.29 ± 3.3 mg/L, total potassium (TK): 815.3 ± 37.9 mg/L, CODcr: 5151.5 ± 274.5 mg/L, Cu: 1.84 ± 0.07 mg/L, Zn: 3.96 ± 0.11 mg/L, Pb: 0.10 ± 0.02 mg/L, and Cd: 0.02 ± 0.01 mg/L.

### Soil Sampling and Chemical Analysis

Soil samples were collected on November 13, 2017, after the rice harvest, when the weather in Shanghai was relatively dry (no rainfall) and the temperature was generally stable (12–18°C). The top-layer soil samples (0–20 cm) were randomly collected with a soil sampler from 10 points in each replicate and thoroughly mixed into a single sample. The soil samples were stored in sterilized, sealed polyethylene bags and immediately transported to the laboratory. One portion of the samples was freeze dried for soil property measurement, and the other portion was stored at −80°C for DNA extraction and high-throughput sequencing analysis.

Soil pH was determined with a pH meter (with a soil:water ratio of 2.5:1); SOC and dissolved organic carbon (DOC) were simultaneously measured with a multi N/C 3,000 total organic carbon analyzer (Analytik Jena AG, Germany); soil TN and available nitrogen (AN) were determined according to the Kjeldahl method (extracted by 2 M KCl); soil TP was determined using the molybdenum-blue colorimetry method (dissolved by 5 M H_2_SO_4_), and available phosphorus (AP) was determined using the molybdenum blue method (extracted by 0.5 M NaHCO_3_); Cu, Zn, Cd, and Pb were measured with an atomic absorption spectrophotometer (AA6880, Shimadzu, Japan).

### High-Throughput Sequencing

Total genomic DNA was extracted from 0.5 g of soil using the PowerSoil^®^ DNA Isolation Kit (12888, MoBio, United States) according to the manufacturer’s instructions. The concentration and quality of the extracted DNA was measured with a K5500 Micro-Spectrophotometer (Beijing, China). The bacterium-biased primer sets 515F (5′-GTGCCAGCMGCCGCGG-3′) and 907R (5′-CCGTCAATTCMTTTRAGTTT-3′) were used for amplification of V3-V4 variance fragments of 16S rRNA (Zhou et al., 2011). The fungus-specific primer set ITS5 (5′-GGAAGTAAAAGTCGTAACAAGG-3′) and ITS4 (5′-TCCTCCGCTTATTGATATGC-3′) was applied for the amplification of the fungal gene in the ITS1 regions (Schoch et al., 2012).

The PCR amplifications were performed using an Applied Biosystems ABI StepOnePlus Real-Time PCR instrument under the following cycling conditions: for the 16S V4-V5 rRNA genes, 30 s denaturation at 98°C, followed by 25 cycles of 98°C for 15 s, 50°C for 30 s, and 72°C for 30 s, and an extension at 72°C for 7 min; for the ITS1 genes, 30 s of initial denaturation at 98°C, followed by 35 cycles of 98°C for 15 s, 50°C for 30 s, and 72°C for 30 s, and an extension at 72°C for 7 min. The PCR products were checked with a NanoDrop ND-1000 UV-Vis spectrophotometer. Sequencing was performed on an Illumina MiSeq platform at Personal Biotechnology Co., Ltd. (Shanghai, China).

Raw sequences were subjected to quality control and assigned to unique 10-bp barcodes using QIIME software (version 1.7.0). The remaining sequences were clustered into operational taxonomic units (OTUs) at the level of 97% sequence similarity. Then, the sequences were annotated through BLAST with the RDP and Unite databases (for bacteria and fungi) using QIIME software (Caporaso and Gordon, 2011).

### Statistical Analysis

The bacterial and fungal richness, diversity and evenness indices (Chao1, abundance-based coverage estimator (ACE), and Shannon and Simpson indices) were calculated using Mothur (version v.1.30.1). One-way analyses of variance (ANOVA) with Tukey’s HSD tests was performed to identify significant differences among the treatments using SPSS 19.0 (SPSS IBM Corp). Partial least squares discriminant analysis (PLS-DA) was performed to detect the bacterial and fungal community differences among the treatments based on the OTU results using R software (version 3.5.1^1^). The species leading to significant differences in the bacterial and fungal communities were revealed using linear discriminant analysis (LDA) effect size (LEfSe). Data on soil bacterial and fungal community structure were used to test the effects of fertilization and biogas slurry application using permutational multivariate analysis of variance (PERMAVONA). In this study, the 25 most abundant bacterial and fungal OTUs from the soil samples were used to construct the networks. The data used in network construction conformed to irhoi >0.6 and *P* < 0.01 in the Spearman correlation analysis, and the network plots were generated using R (version 3.5.1, iGraph and qgraph packages).

## Results

### Soil Property Characteristics and Rice Yields

Compared to the values in RT, all the treatments involving biogas slurry combined with chemical fertilizer significantly increased the SOC, DOC, SOC/DOC, AN and AP by 7.53, 53.30, 42.59, 11.97, and 7.62% on average, respectively, and significantly decreased the pH by 0.19 on average (Table 2). The contents of DOC, AN, and AP were the highest under the treatments BS50 and BS75. In addition, SOC under BS50 and SOC/DOC under BS75 were significantly higher than those in the other treatments. The soil TN and TP contents showed no significant differences among the treatments.

**TABLE 2:** Soil properties associated with the rice harvest after 5 years of treatment application.

**Treatment**	**pH**	**SOC g^.^kg^–1^**	**DOC mg^.^kg^–1^**	**DOC/SOC %**	**TN g^.^kg^–1^**	**TP g^.^kg^–1^**	**AN mg^.^kg^–1^**	**AP mg^.^kg^–1^**
CK	8.63 ± 0.09c	8.41 ± 0.45c	47.38 ± 2.05c	5.63 ± 0.45c	0.92 ± 0.09b	0.84 ± 0.08b	53.61 ± 2.02d	16.75 ± 0.73d
RT	8.81 ± 0.07a	8.32 ± 0.40c	39.84 ± 1.86d	4.79 ± 0.39d	1.05 ± 0.11a	0.95 ± 0.09a	57.19 ± 1.81c	17.33 ± 0.82c
BS25	8.76 ± 0.04b	8.75 ± 0.27b	58.60 ± 2.37b	6.70 ± 0.58b	1.04 ± 0.10a	0.94 ± 0.12a	59.57 ± 1.92b	17.92 ± 0.74b
BS50	8.55 ± 0.08d	9.47 ± 0.42a	63.68 ± 2.44a	6.72 ± 0.61b	1.03 ± 0.12a	0.93 ± 0.10a	65.31 ± 2.33a	18.85 ± 0.93a
BS75	8.56 ± 0.06d	8.62 ± 0.32b	60.95 ± 1.96a	7.07 ± 0.53a	1.04 ± 0.09a	0.94 ± 0.07a	67.23 ± 2.14a	19.18 ± 0.80a
BS100	8.66 ± 0.09c	8.57 ± 0.53bc	57.94 ± 2.18b	6.76 ± 0.51b	1.05 ± 0.10a	0.93 ± 0.06a	60.36 ± 1.87b	18.09 ± 0.64b

*SOC, soil organic C; DOC, dissolved organic C; TN, total nitrogen; TP, total phosphorus; AN, available nitrogen; AP, available phosphorus.*

*Values in the table are the mean ± SE; different letters in the same column indicate a significant difference (*P* < 0.05).*

**FIGURE 1:**
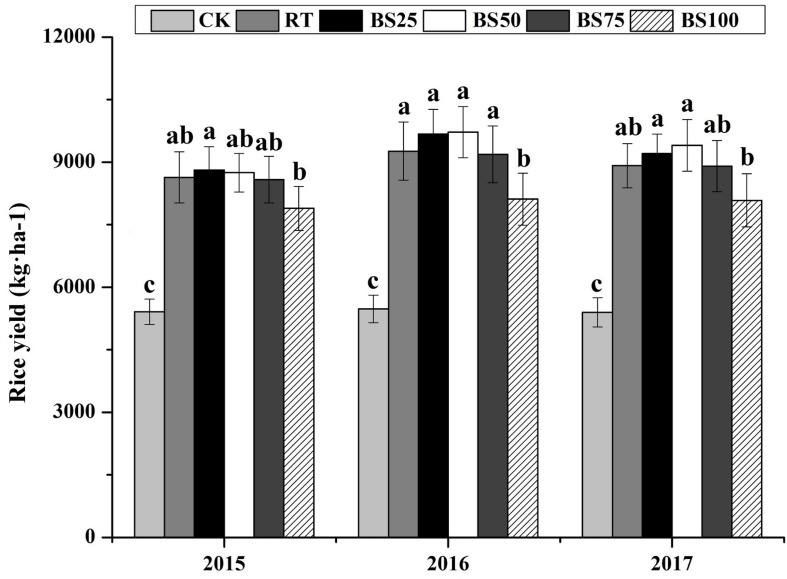
Rice yields in different treatments during 2015–2017. Different letters above the columns indicate significant differences at *P* < 0.05.

In contrast, there was no significant difference between RT and the treatments of biogas slurry combined with chemical fertilizer in terms of rice yield over the 3 years (Figure 1). Compared with that under the BS100 treatment, biogas slurry combined with chemical fertilizer increased the rice yield by 8.75–11.67%, 13.25–19.82%, and 10.11–16.30% in 2015, 2016, and 2017, respectively.

### Bacterial and Fungal α Diversity and the Correlation With Soil Properties

Bacterial and fungal α diversity was notably affected by the different fertilization regimes. Chao1 and ACE were used as richness indices, and the Shannon index was employed as the diversity index. Chao1 and ACE for the bacterial community under the treatments with biogas slurry were significantly higher than those under RT, and BS50 and BS75 had the highest values (Table 3). The Shannon index of the bacterial community under BS50 and BS75 was also significantly higher than that under RT (*P* < 0.05). In contrast, compared with RT, the treatments with biogas slurry significantly decreased the Chao1, ACE and Shannon index values for the fungal community, and BS50 and BS75 had significantly higher values of these indices than BS25 and BS100 (*P* < 0.05).

**TABLE 3:** Richness and diversity indices of bacteria and fungi in different treatments.

	**Treatment**	**Chao1**	**ACE**	**Shannon**
Bacteria	CK	2496.03 ± 224.62c	2840.01 ± 273.02c	10.38 ± 0.07c
	RT	2561.64 ± 176.64c	2874.86 ± 189.86c	10.38 ± 0.11c
	BS25	3052.13 ± 294.05b	3108.70 ± 257.39b	10.45 ± 0.03b
	BS50	3201.37 ± 114.02a	3412.32 ± 157.18a	10.53 ± 0.10a
	BS75	3329.44 ± 140.25a	3496.03 ± 225.41a	10.55 ± 0.13a
	BS100	3105.08 ± 168.38b	3287.81 ± 159.78b	10.48 ± 0.09ab
Fungi	CK	603.02 ± 63.95d	603.12 ± 72.42d	5.31 ± 0.32c
	RT	921.88 ± 52.77a	927.46 ± 47.68a	6.24 ± 0.28a
	BS25	720.34 ± 85.34c	722.29 ± 57.29c	5.38 ± 0.49c
	BS50	781.14 ± 77.74b	769.76 ± 81.82b	5.78 ± 0.33b
	BS75	765.77 ± 73.77b	760.15 ± 88.15b	6.01 ± 0.46b
	BS100	708.72 ± 66.96c	707.59 ± 74.69c	5.35 ± 0.35c

*Values in the table are the mean ± SE; different letters in the same column indicate a significant difference (*P* < 0.05).*

The results of the correlation analysis between the soil properties and the bacterial and fungal richness and diversity indices are shown in Table 4. The Chao1, ACE and Shannon index values of the bacterial community were significantly positively correlated with the DOC, DOC/SOC, AN, and AP. However, there were no significant correlations between the soil properties and the richness and diversity index values of the fungal community.

**TABLE 4:** Correlations between soil properties and microbial richness and diversity indices.

	**Bacteria**			**Fungi**		
**Items**	**Chao1**	**ACE**	**Shannon**	**Chao1**	**ACE**	**Shannon**
pH	–0.509	–0.648	–0.675	0.402	0.450	0.102
SOC	0.561	0.578	0.612	0.018	–0.029	–0.034
DOC	0.843**	0.805*	0.820*	–0.281	–0.323	–0.272
DOC/SOC	0.841**	0.787*	0.794*	–0.362	–0.396	–0.333
TN	0.539	0.471	0.425	0.646	0.637	0.401
TP	0.504	0.432	0.399	0.715	0.706	0.501
AN	0.842**	0.876**	0.883**	0.257	0.212	0.356
AP	0.869**	0.892**	0.895**	0.235	0.191	0.312

**Significant at the 0.01 level (*P* < 0.01), *significant at the 0.05 level (*P* < 0.05).

### Bacterial and Fungal Community Composition

**FIGURE 2:**
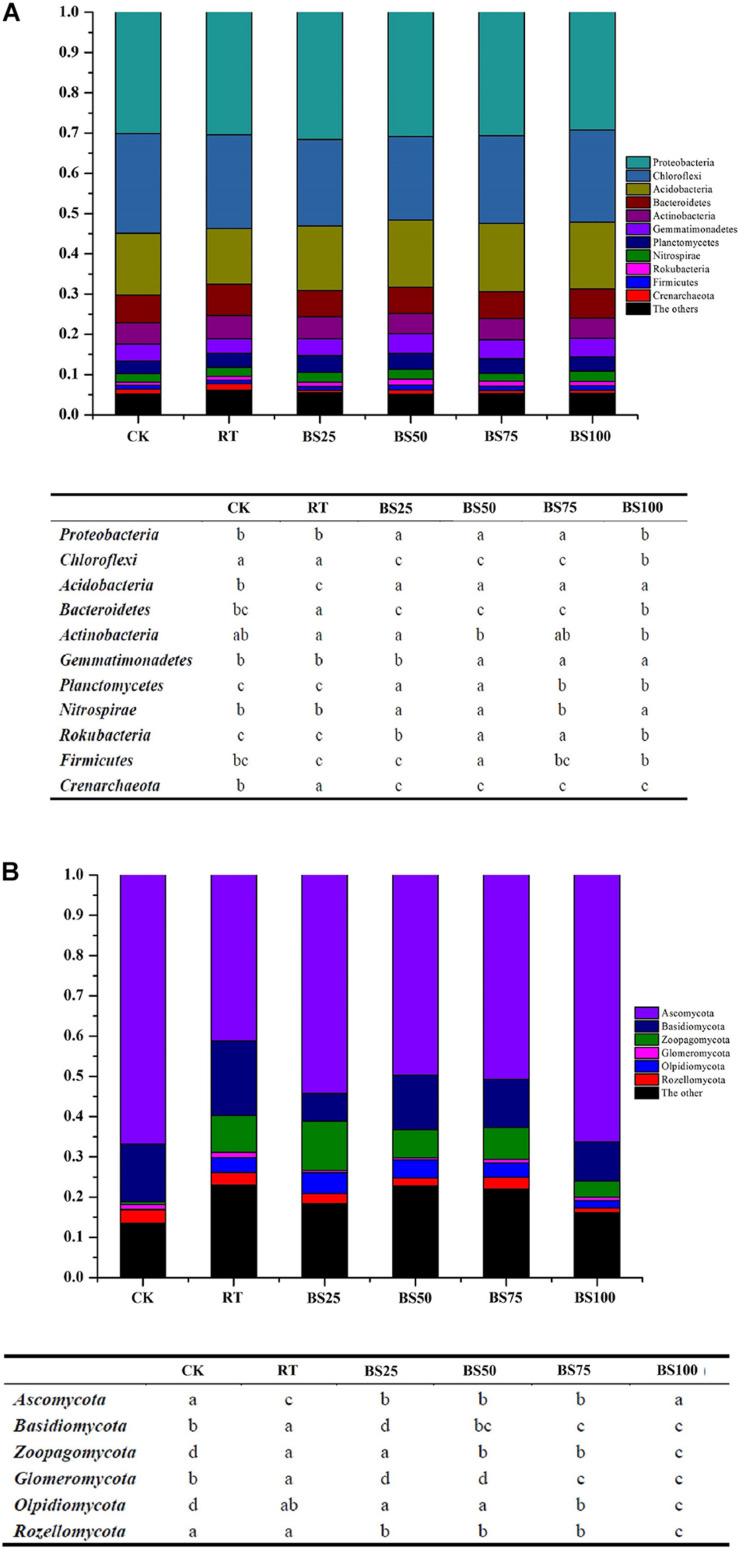
Phylogenetic classification of dominant bacterial **(A)** and fungal **(B)** phyla and their summarized phyla with statistical differences in different treatments. Different letters at each phylum indicate significant differences at *P* < 0.05 according to the LSD test.

The relative abundances of the main soil bacterial taxa are shown in Figure 2A. *Proteobacteria*, *Chloroflexi*, and *Acidobacteria* (in successive order) were the dominant phyla in the paddy soil across all treatments, accounting for 67.52–70.22% of the total bacterial sequence data. The other phyla with substantial relative abundances (1–10%) were *Bacteroidetes* (6.46–7.82%), *Actinobacteria* (5.04–5.80%), *Gemmatimonadetes* (3.55–4.83%), *Planctomycetes* (3.12–4.12%), *Nitrospirae* (1.91–2.54%), *Rokubacteria* (0.74–1.43%), *Firmicutes* (0.9–1.26%) and *Crenarchaeota* (0.59–1.61%). Among these, compared to those in RT, the relative abundances of *Proteobacteria*, *Acidobacteria*, *Planctomycetes*, and *Rokubacteria* significantly increased (*P* < 0.05) by 0.82–3.82%, 16.34–22.92%, 5.73–18.05%, and 15.22–56.52%, respectively, under the treatments involving biogas slurry combined with chemical fertilizer, while the relative abundances of *Chloroflexi*, *Bacteroidetes*, and *Crenarchaeota* were significantly reduced (*P* < 0.05) by 7.77–10.82%, 14.19–17.39%, and 37.27–63.35%, respectively.

The relative abundances of the main soil fungal taxa are shown in Figure 2B. *Ascomycota*, *Basidiomycota*, and *Zoopagomycota* (in successive order) were the dominant phyla in the paddy soil across all treatments, accounting for 68.91–81.82% of the total fungal sequence data. The other phyla with substantial relative abundances (1–10%) were *Olpidiomycota* (0.24–5.19%), *Rozellomycota* (1.16–3.27%), and *Glomeromycota* (0.52–1.29%). Among these, compared with their relative abundances in RT, the treatments with biogas slurry combined with chemical fertilizer significantly promoted *Ascomycota* by 20.51–31.59% (*P* < 0.05) and significantly decreased (*P* < 0.05) *Basidiomycota*, *Glomeromycota*, and *Rozellomycota* by 26.66–62.41%, 30.23–59.69%, and 6.23–37.05%, respectively.

### Comparison of Bacterial and Fungal Community Composition Among the Different Treatments

**FIGURE 3:**
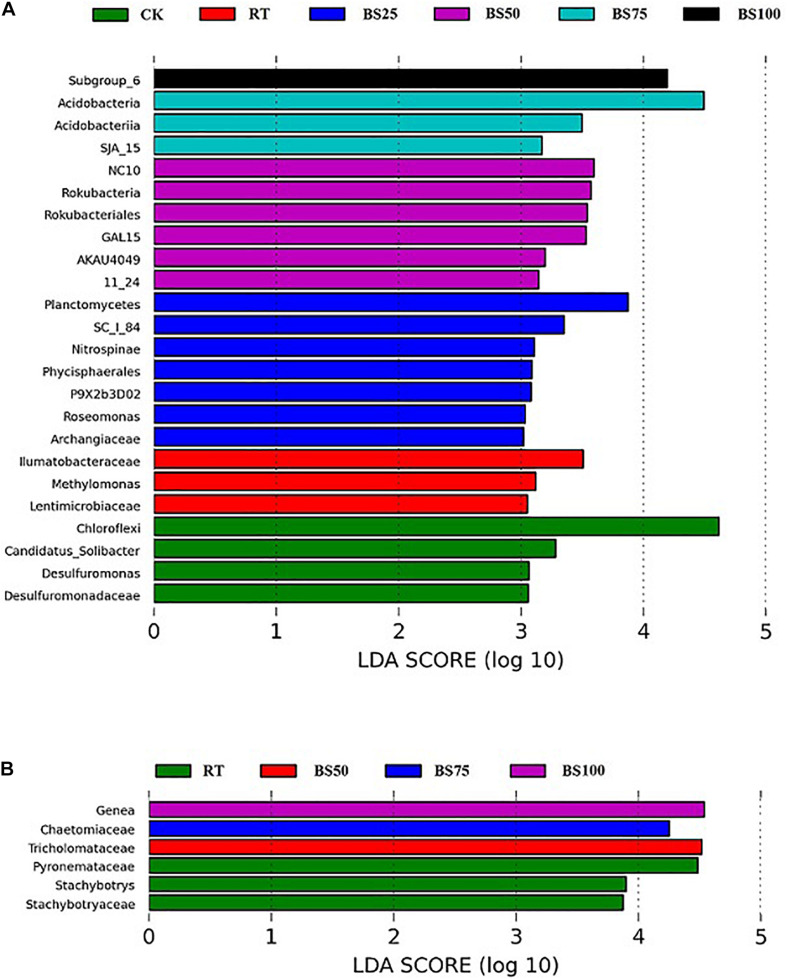
LEfSe analysis of the most differentially abundant bacterial **(A)** and fungal **(B)** taxa in the soil samples (LDA score >3).

Linear discriminant analysis (LDA) was applied to identify differences in bacterial and fungal groups among the different fertilization regimes. Twenty-four bacterial communities had LDA scores greater than 3 (Figure 3A). The distribution of bacterial communities with significant phylogenetic differences was as follows: 6 at the phylum level, 5 at the class level, 4 at the order level, 5 at the family level and 4 at the genus level. Among these, 16 communities were from the treatments including biogas slurry combined with chemical fertilizer. Only 3 communities had LDA scores greater than 4, which were *Subgroup_6* (class of *Acidobacteria*) under BS100, *Acidobacteria* under BS75 and *Chloroflexi* under CK. Six fungal communities had LDA scores greater than 3 (Figure 3B). The distribution of fungal communities with significant phylogenetic differences was 4 at the family level and 2 at the genus level. Among these communities, 3 were from RT, which had the highest number. Four fungal taxa had LDA scores greater than 4: *Genea* (genus of *Ascomycota*) under BS100, *Chaetomiaceae* (family of *Ascomycota*) under BS75, *Tricholomataceae* (family of *Basidiomycota*) under BS50 and *Pyronemataceae* (family of *Ascomycota*) under RT.

### Bacterial and Fungal Community Structure and Determinants

**FIGURE 4:**
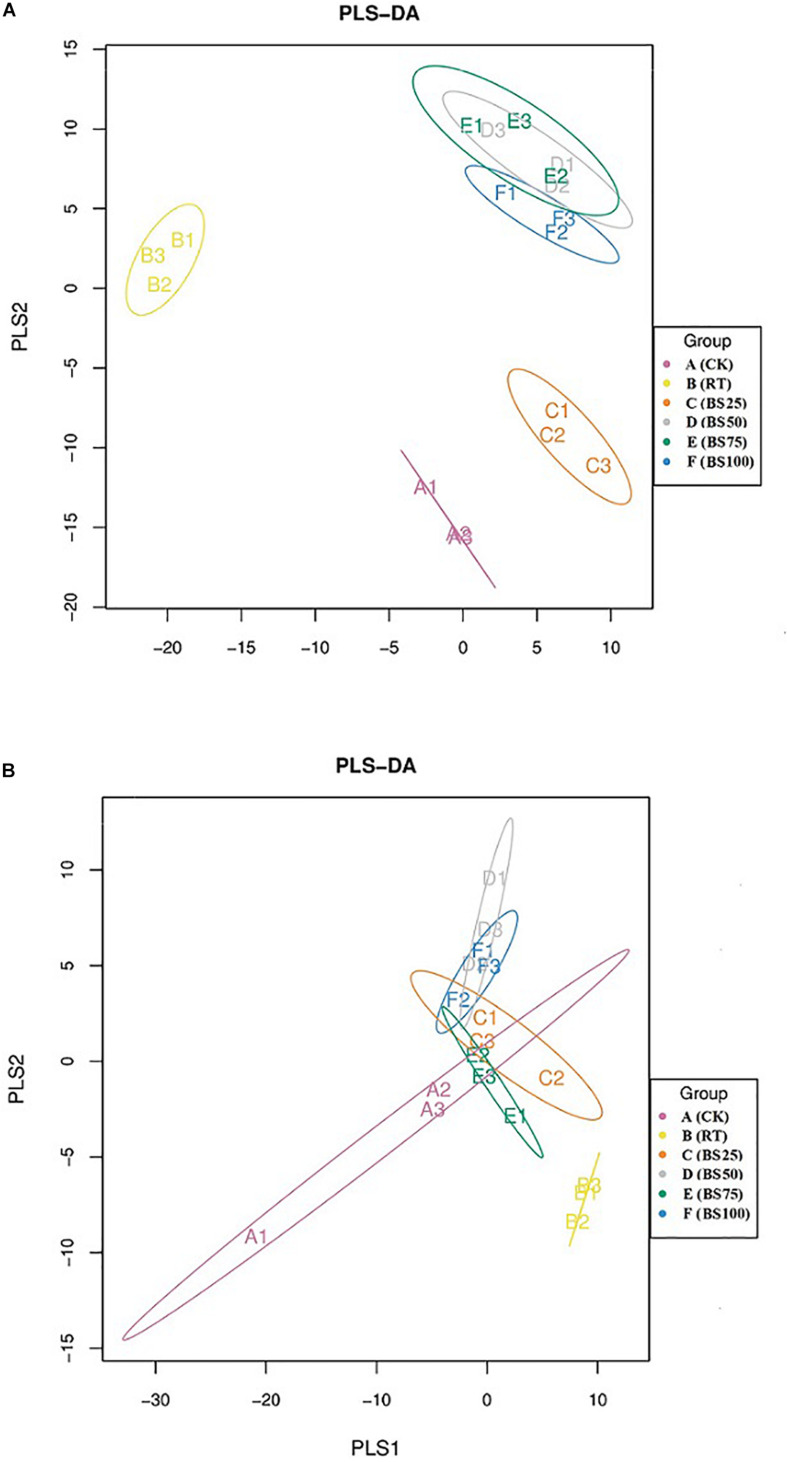
Partial least squares discriminant analysis (PLS-DA) of the soil bacterial **(A)** and fungal **(B)** communities in different treatments.

**FIGURE 5:**
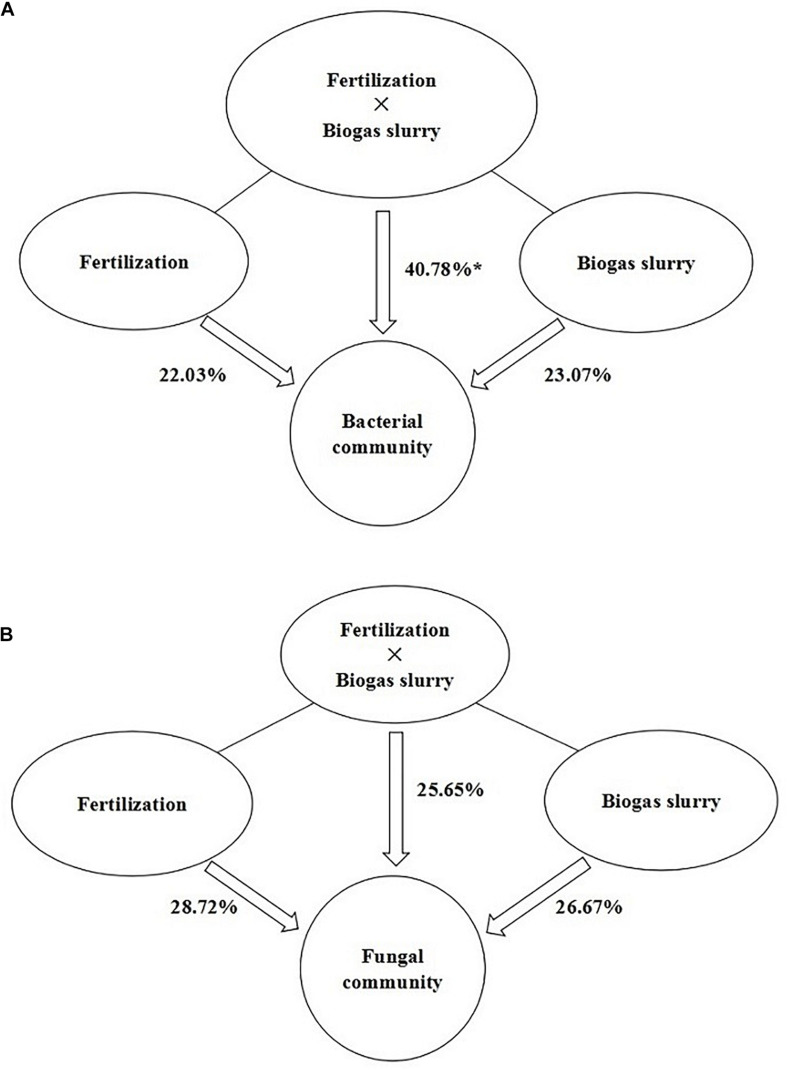
PERMANOVA of the contributions of the application of fertilizer, biogas slurry and fertilizer × biogas slurry to the variation in bacterial **(A)** and fungal **(B)** community compositions. * represents significant differences at *P* < 0.05.

The PLS-DA analysis showed that the composition of bacterial communities under CK and RT was distinctly separated from that under the treatments with biogas slurry along the X axis (Figure 4A), indicating a certain influence of biogas slurry on the bacterial community. The PERMANOVA also confirmed that the bacterial community was more altered by biogas slurry × fertilization than by fertilization or application of biogas slurry alone (Figure 5A). The similarity in bacterial community structure was highest between BS50 and BS75. However, there were no significant differences in the fungal community structure among any of the treatments except RT (Figure 4B). The PERMANOVA results suggest that biogas slurry × fertilization, biogas slurry and fertilization had statistically equal impacts on the fungal community (Figure 5B).

### Network Associations Among Bacterial and Fungal Communities

**FIGURE 6:**
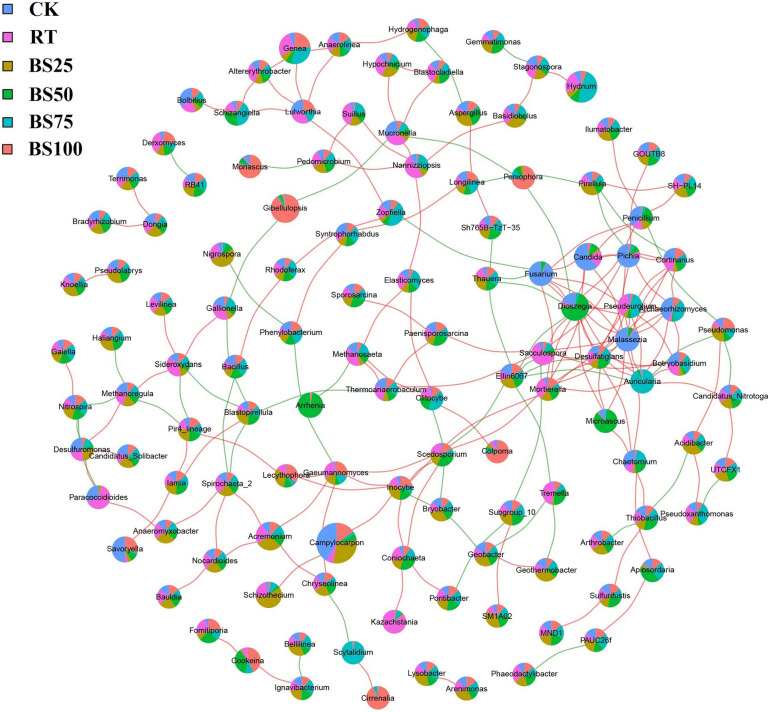
Network analysis based on bacterial and fungal OTUs with the abundance higher than 0.1%. Red and green lines represent significantly positive and negative correlations (*P* < 0.01), respectively. Node size is positively related to OTU abundance. Colored proportions of nodes signify OTU abundance in relation to the different treatments.

Network construction was based on the bacterial and fungal OTUs at the genus level with the abundance higher than 0.1% in the experimental soil (Figure 6). The network consisted of 329 significant associations (edges) among 132 nodes, including 278 positive and 51 negative links, and node size was positively proportionate to its abundance. The top 10 taxa with high number of interactions were *Dioszegia* (genus of *Basidiomycetes*, 17 links), *Malassezia* (genus of *Basidiomycetes*, 14 links), *Auricularia* (genus of *Basidiomycetes*, 12 links), *Pseudeurotium* (genus of *Ascomycetes*, 10 links), *Fusarium* (genus of *Ascomycetes*, 9 links), *Ellin6067* (genus of *Proteobacteria*, 9 links), *Archaeorhizomyces* (genus of *Ascomycetes*, 8 links), *Desulfatiglans* (genus of *Proteobacteria*, 8 links), *Mortierella* (genus of *Zygomycetes*, 8 links), and *Candida* (genus of *Ascomycetes*, 7 links).

## Discussion

### Effects on Soil Nutrient Availability and Production of the Paddy Field

In the present study, compared with RT, all the treatments involving biogas slurry combined with chemical fertilizer significantly increased the SOC and DOC contents and SOC/DOC, indicating that biogas slurry combined with chemical fertilizer could have a positive impact on SOC sequestration, especially in terms of labile organic carbon (Table 2). Similar results were observed in several other studies (Ozores-Hampton et al., 2011; Xu M. et al., 2019, Xu Z. et al., 2019). With the increasing ratio between biogas slurry and chemical fertilizer, the SOC and DOC contents first increased and then decreased, implying that a proper replacement dose (50% in the present study) of biogas slurry best benefits soil organic matter content. This result was also consistent with that found by Zheng et al. (2017), who reported that a pure nitrogen rate of chemical fertilizer to biogas slurry of 0.45:0.55 best improved the soil nitrogen and organic matter content. In the present study, the soil AN and AP under the fertilization treatments had the same pattern as the DOC, but there were no differences in the TN and TP among the fertilization treatments, showing that biogas slurry significantly elevated the soil nutrient availability, especially when biogas slurry was applied with chemical fertilizer. This is in agreement with the results of an experiment involving biogas slurry application in peanut cultivation performed by Zheng et al. (2015), who found that the treatment with 30% pure nitrogen replaced by biogas slurry enhanced the soil-available nutrients and crop yield.

In the present study, compared with that under RT, biogas slurry combined with chemical fertilizer did not statistically promote paddy yield (Figure 1). However, the average of paddy yields under BS25 and BS50 were higher than those under RT during the 3 years, and there was a trend of the growing difference between RT and BS50 being 1.31–5.48%. Biogas slurry contains various nutrients, such as organic matter, N, P, K, Mg, and Ca, and can be considered a liquid organic fertilizer. Under the same pure nitrogen conditions, biogas slurry combined with chemical fertilizer provided both inorganic nutrients and organic nutrients. An appropriate fertilizer doses and a proper ratio of biogas slurry and chemical fertilizer could offer nutrients for crop growth in a timely manner and reduce carbon and nitrogen losses caused by leaching and gas emissions, consequently increasing crop yield (Abubaker et al., 2012; Win et al., 2014; Cheng et al., 2018). In the present study, BS50 was found to be better in terms of the soil nutrient availability and the production of paddy fields than the other treatments.

### Effects on Microbial α Diversity and Its Relationships With Soil Properties

Our results showed that compared with RT, all the treatments with biogas slurry enhanced the bacterial α diversity and that the values under BS50 and BS75 were the highest, showing a significant positive impact of biogas slurry on the bacterial community, especially when it was combined with chemical fertilizer (Table 3). This was likely due to the extensive organic matter within the biogas slurry and the ability of the biogas slurry combined with chemical fertilizer to elevate the content of soil available nutrients, which could also promote the growth of the bacterial community (Johansen et al., 2013; Zheng et al., 2015). Correlation analysis showed that the DOC, DOC/SOC, AN, and AP were positively correlated with the bacterial α diversity (Table 4); these variables are all soil availability indices, further confirming the significance of soil available nutrients to bacterial α diversity. Biogas slurry significantly elevated the soil nutrient availability, consequently raised the bacterial α diversity, showing the indirect positive correlation between bacterial community and biogas slurry, especially when biogas slurry applied with chemical fertilizer. This correlation result is also in agreement with Xu M. et al. (2019); Xu Z. et al. (2019), who reported that richness and diversity index values were positively correlated with AN, AP, and SOM.

In the present study, compared with that under RT, all the treatments with biogas slurry weakened the fungal α diversity, and the values under BS25 and BS100 were the lowest (Table 3), indicating an inhibitory effect of biogas slurry on the fungal community. Geisseler and Scow (2014) reviewed 64 long-term experiments and claimed that in general, mineral fertilizer is more likely to cause fungal growth than organic fertilizer. A higher concentration of soil available nutrients may be responsible for the negative effects on the fungal community by restraining the colonization of arbuscular mycorrhizal fungi (Wentzel and Joergensen, 2016). The bacterial community was also apt to thrive under the high available nutrient conditions, which gave them some advantage in the interaction with the fungal community (Walsh et al., 2012). Although the fungal community was altered by some soil conditions, there was no strong correlation between the fungal α-diversity indices and soil properties (Table 4). TN and TP had a higher correlation than the other properties, but it was not significant, implying that the effective factors of the fungal community were more complex and diverse than those of the bacterial community (Wang H. et al., 2018).

### Effects on Bacterial Community Composition

With the increasing ratio between biogas slurry and chemical fertilizer, the abundance of *Proteobacteria* first increased and then decreased. This was similar to the observations of Xu M. et al. (2019), Xu Z. et al. (2019). *Proteobacteria* have been demonstrated to have the ability to degrade recalcitrant organic compounds and could be promoted by a high liable organic carbon content (Goldfarb et al., 2011; Hamm et al., 2016), suggesting that biogas slurry combined with chemical fertilizer improved the cycling of organic matter. The abundances of *Planctomycetes* and *Rokubacteria* under the treatments with biogas slurry addition were all higher than under RT. Previous studies have shown that *Planctomycetes* play an important role in carbon utilization and have the potential to generate antibiotics to protect their habitat (Jeske et al., 2016). *Rokubacteria* is considered to contribute to carbon metabolism in a wide range of gaseous and solid substrates (Kroeger et al., 2018). The relative abundances of *Acidobacteria* under biogas slurry addition were higher than those under RT by 16.34–22.92%, which was likely caused by *Acidobacteria* preferring high amounts of carbon and diverse carbon sources (Xu et al., 2016). In addition, *Nitrospirae* relative abundance was significantly higher under BS25 than under the other treatments (Figure 3A). *Nitrospirae* has a high nitrogen metabolism efficiency and might also exert a positive effect on carbon fixation and crop yield (Pachiadaki et al., 2017). *Chloroflexi* and *Crenarchaeota* were clearly inhibited by biogas slurry application in the present study. *Chloroflexi*, widely reported as heterotrophic oligotroph and facultative anaerobic bacteria, are sensitive to soil pH and thrive at neutral pH (Hug et al., 2013). Some members of *Crenarchaeota* play a key role in carbon, nitrogen, and sulfur biochemical cycling (Wang H. et al., 2018). The abundance of *Crenarchaeota* was observed to decline in a nitrogen-rich environment in a previous study, likely owing to the autotrophic growth ability of the members of this taxon (Bates et al., 2011).

The results of the network association analysis (Figure 6) showed that there were only 2 bacterial taxa in the top 10 taxa with high number of interactions. *Ellin6067* (2 positive and 7 negative links) has been considered as an ammonia-oxidizing bacterium, involved in soil nitrification process (Ye et al., 2016). BS25 had the highest abundance of *Ellin6067* among all the treatments, implying such mixed applications might be beneficial to soil nitrogen cycling. *Desulfatiglans* (7 positive and 1 negative links) play important roles in soil sulfur cycling (Wang et al., 2019), and was most enriched under BS50, showing the ability of BS50 to promote sulfur nutrient circulation.

### Effects on Fungal Community Composition

*Ascomycota* was the most abundant and dominant phylum in the fungal community, accounting for 41.19–66.83% of all sequences (Figure 2B), which was consistent with some previous studies in paddy soil (Liu et al., 2016; Wang H. et al., 2018). This phylum is known as an important decomposer of lignocellulose organic matter (Wang et al., 2015). Compared with that under RT, biogas addition significantly enriched the abundance of *Ascomycota*, showing a positive impact of biogas slurry on soil organic matter decomposition, especially on lignocellulose organic matter. Moreover, the abundance of not all *Ascomycota* species was increased by biogas slurry addition, and the LEfSe analysis (Figure 3B) showed that *Stachybotrys* (genus of *Ascomycota*) and *Stachybotryaceae* (family of *Ascomycota*) significantly increased in abundance under RT. *Stachybotrys* generates diverse toxins, such as satratoxins and atranones, which have toxic effects on human health (Semeiks et al., 2014). Some species of *Stachybotryaceae* are saprophytes and weak pathogens of certain plants and can also endanger human health through the air (Liang et al., 2019). This demonstrates that RT raised the possibility of some potential diseases, while biogas slurry addition can inhibit such diseases to some extent. Two phyla (*Basidiomycota* and *Glomeromycota*) were significantly depleted by biogas slurry application in the present study. *Basidiomycota* are oligotrophs and adapted to relatively poor-nutrient environments (Sun et al., 2018); *Glomeromycota* are capable of assimilating nutrients directly from the environment and are enriched with decreasing carbon and nitrogen levels (Zhang et al., 2019). The species of *Zoopagomycota* are mainly parasites and pathogens of small animals and some fungi (Spatafora et al., 2016). Compared with that under RT, the abundance of *Zoopagomycota* was decreased by all treatments involving biogas slurry addition except BS25, which showed no significant difference with RT. The results indicate that biogas slurry addition might have the ability to control *Zoopagomycota* pathogens, but the effect also depends on the pure nitrogen ratio of biogas slurry and chemical fertilizer (higher than 1:3 in the present study).

*Dioszegia* are epiphytic phylloplane yeasts and play a key role in plant-microorganism relationships (Takashima et al., 2019). According to the network association analysis (Figure 6), *Dioszegia* had 14 positive and 3 negative links, and BS75 promoted *Dioszegia* the most. *Pseudeurotium* (10 positive links) and *Archaeorhizomyces* (8 positive links) were both significantly enriched under BS75. *Pseudeurotium* have been reported to have an impact on crop growth promotion and pathogen inhibition (Zhou et al., 2018), and members of *Archaeorhizomyces* are considered to have the potential to promote the growth of grasslands (Zhang et al., 2018). The genus *Auricularia* (10 positive and 2 negative links) usually grows on wood and plays an important role in human food supply and nutrition (Dai et al., 2019), which was also significantly promoted by BS75. BS75 demonstrated prominent positive influences of crop growth and pathogen inhibition in the network association analysis.

## Conclusion

In conclusion, on the basis of maintaining a stable paddy yield, 3 years of the application of biogas slurry combined with chemical fertilizer significantly improved soil properties and affected bacterial and fungal community diversity and composition. Compared with those under regular chemical fertilization, biogas slurry combined with chemical fertilizer significantly increased the bacterial diversity and decreased the fungal diversity. Bacterial community was promoted by DOC, DOC/SOC, AN, and AP, but no soil properties were significantly associated with the fungal community. Biogas slurry combined with chemical fertilizer had a significant impact on the bacterial community and had roughly equal influences on the fungal community as pure biogas slurry and chemical fertilizer application. The observation of some changes in specific microbial taxa caused by the application of biogas slurry indicates that the addition of biogas slurry exerted both positive and negative impact on plant growth and soil fertility, but a proper ratio of biogas slurry and chemical fertilizer may have a positive effect on crop growth and pathogen control.

## Data Availability Statement

The datasets presented in this study can be found in online repositories. The names of the repository/repositories and accession number(s) can be found below: NCBI, PRJNA623237 (https://www.ncbi.nlm.nih.gov/sra/PRJNA623237).

## Author Contributions

HanZ contributed to analysis, interpretation of data, and writing original draft of the manuscript. WL contributed to the conception and design of the study. SL and XZ contributed to field investigation and sample acquisition. NB, JZ, and HaiZ contributed to the editing and revising of the manuscript. All authors contributed to the article and approved the submitted version.

## Conflict of Interest

The authors declare that the research was conducted in the absence of any commercial or financial relationships that could be construed as a potential conflict of interest.

## Publisher’s Note

All claims expressed in this article are solely those of the authors and do not necessarily represent those of their affiliated organizations, or those of the publisher, the editors and the reviewers. Any product that may be evaluated in this article, or claim that may be made by its manufacturer, is not guaranteed or endorsed by the publisher.

## Footnotes

1
http://www.r-project.org/
